# A Detailed Protocol for Constructing a Human Single-Chain Variable Fragment (scFv) Library and Downstream Screening via Phage Display

**DOI:** 10.3390/mps7010013

**Published:** 2024-02-01

**Authors:** Ziyi Liu, Dokyun Kim, Seokmin Kang, Jae U. Jung

**Affiliations:** 1 Cancer Biology Department, Infection Biology Program, Lerner Research Institute, Cleveland Clinic, Cleveland, OH 44195, USA; liuz5@ccf.org (Z.L.); kimd9@ccf.org (D.K.); kangs5@ccf.org (S.K.); 2 Global Center for Pathogen Research and Human Health, Lerner Research Institute, Cleveland Clinic, Cleveland, OH 44195, USA

**Keywords:** single-chain variable fragment (scFv), phage display library, antibody screening

## Abstract

The development of monoclonal antibodies (mAbs) represents a significant milestone in both basic research and clinical applications due to their target specificity and versatility in therapeutic and diagnostic applications. The innovative strategy of mAb screening, utilizing phage display, facilitates the in vitro screening of antibodies with high affinity to target antigens. The single-chain variable fragment (scFv) is a subset of mAb derivatives, known for its high binding affinity and smaller size—just one-third of that of human IgG. This report outlines a detailed and comprehensive procedure for constructing a scFv phagemid library derived from human patients, followed by screening via phage display affinity selection. The protocol utilizes 348 primer combinations spanning the entire human antibody repertoire to minimize sequence bias and maintain library diversity during polymerase chain reaction (PCR) for scFv generation, resulting in a library size greater than 1 × 10^8^. Furthermore, we describe a high-throughput phage display screening protocol using enzyme-linked immunosorbent assay (ELISA) to evaluate more than 1200 scFv candidates. The generation of a highly diverse scFv library, coupled with the implementation of a phage display screening methodology, is expected to provide a valuable resource for researchers in pursuit of scFvs with high affinity for target antigens, thus advancing both research and clinical endeavors.

## 1. Introduction

Monoclonal antibodies (mAbs) are pivotal in immunotherapeutic strategies for a broad spectrum of diseases, including autoimmune disorders, chronic inflammatory diseases, cancer, and infectious diseases [1,2]. Their clinical efficacy is evidenced by the success of Trastuzumab (Herceptin) in treating HER2-positive breast cancer [3], Adalimumab (Humira) for chronic rheumatoid arthritis and ulcerative colitis [4], and the Tixagevimab/Cilgavimab cocktail (Evusheld) targeting the SARS-CoV-2 Spike protein to combat COVID-19 [5]. Currently, there are more than 140 FDA-approved or regulatory-reviewed mAb-based drugs in the United States alone [2,6].

**Figure 1:**
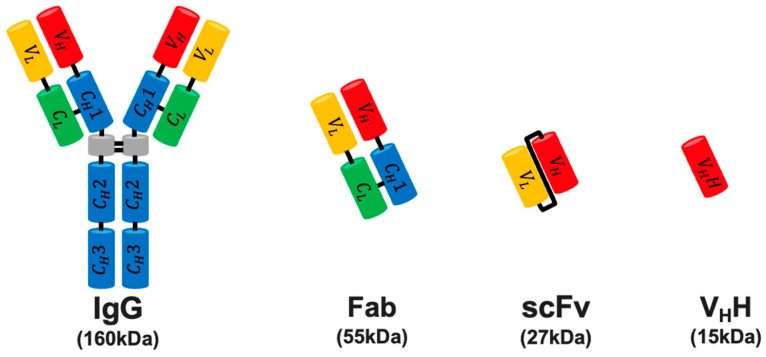
mAb and its derivatives. IgG is composed of two identical heavy chains (V_H_) and two light chains (V_L_), where the light chains are either kappa (κ) or lambda (λ) light chains. Antibody derivatives are notably smaller than IgG. The scFv includes only the variable regions, comprising the antigen-binding domain.

Motivated by this clinical success, derivatives of mAbs have been developed to expand the utilization of mAb-based diagnostics and therapeutics for various diseases, while preserving their target specificity and high binding affinity [7]. The conventional antibody, or immunoglobulin (Ig), is a “Y”-shaped tetrameric protein, formed by two heavy-chain–light-chain heterodimers connected by disulfide bonds (Figure 1). Comprising three functional domains, it includes two identical antigen-binding fragments (Fabs) and one crystallizable fragment (Fc). The variable fragment (Fv) located at the tip of the variable regions of the heavy chain (V_H_) and light chain (V_L_) is responsible for mediating target specificity. The combination of various kappa (κ) or lambda (λ) light chains with the heavy chain confers specificity against a wide range of antigens with high binding affinity. Consequently, V_H_ and V_L_ have been rearranged into diverse formats, including Fab, single-chain Fv (scFv), and variable heavy domains of heavy chain (V_H_H) antibodies [8].

scFv, first developed by Bird et al. in 1988 [9], is a recombinant polypeptide where V_H_ is joined to V_L_ via a flexible linker composed of glycine and serine amino acids [10]. With a molecular weight of approximately 27 kDa, scFvs are much smaller than conventional whole Ig-based monoclonal antibodies (~150 kDa), enabling more effective tissue penetration and access to epitope crevices, while retaining high antigen-binding affinity [11]. Their rapid systemic clearance has led to widespread use in therapeutic and imaging applications in clinical settings. Furthermore, the lack of an Fc region diminishes the immunogenicity of scFvs, broadening their therapeutic application scope. Additionally, their cost-effective bacterial production enhances the accessibility of scFvs as antibody-based therapeutics [10]. Due to these benefits, scFvs are increasingly utilized in clinical scenarios where conventional Ig and Fab are suboptimal, such as in Chimeric Antigen Receptor (CAR) T-cell therapy [12].

The discovery of antibodies involves genetic manipulation of recombinant antibody fragments. Historically, antibody development was revolutionized by hybridoma technology, established by Köhler and Milstein in 1975, enabling large-scale production of specific antibodies [13]. Nevertheless, this technique has limitations. For instance, most mAbs produced via hybridoma technology are of murine origin and may induce human anti-mouse antibody responses in clinical settings [14]. To mitigate immunogenicity, antibodies from non-human species undergo a humanization process, either through chimerization or by grafting complementarity-determining regions (CDRs) on scaffolds from human Ig. However, these modifications can compromise the antibodies’ binding affinity or stability, potentially reducing their therapeutic efficacy [15,16]. Consequently, screening antibodies from a human library is deemed the most effective approach for developing clinically applicable antibodies [17].

Among various antibody libraries, such as synthetic, semi-synthetic, naïve, immune libraries, and predefined CDR libraries tailored to specific requirements, the immune library offers a distinct advantage due to its repertoire specifically elicited against the target antigen [17,18,19,20,21]. Affinity maturation through somatic hypermutation and repeated exposure via immunization or natural infection enhances the specificity and efficacy of the antibodies. Antibodies sourced from immune libraries exhibit superior binding affinities compared to those from synthetic or naïve libraries [17,22]. The human immune library, boasting its inherent diversity and eliminating the need for humanization, is an ideal source for library construction. Nonetheless, constructing a human immune library presents challenges, such as ethical considerations and the impracticality of deliberate immunization with the target antigen. Hence, the creation of a human immune scFv library typically relies on donated samples from individuals naturally exposed to the antigen or infected with the target pathogen [17,18,20,23].

The innovative development of phage display by George P. Smith has revolutionized the field, offering an efficient technique for generating antibodies against specific antigens [24]. Phage display screening, or affinity selection, is characterized by its rapidity, the ability to screen vast libraries, superior efficiency compared to conventional hybridoma methods, and cost-effectiveness [25,26,27]. This method allows for the display of diverse scFv repertoires on the surface of filamentous phages, facilitating their binding to the desired antigen. Through repeated selection rounds, an in vitro evolution of the displayed antibodies occurs, enriching for scFvs with high affinity for the antigen [28].

**Figure 2:**
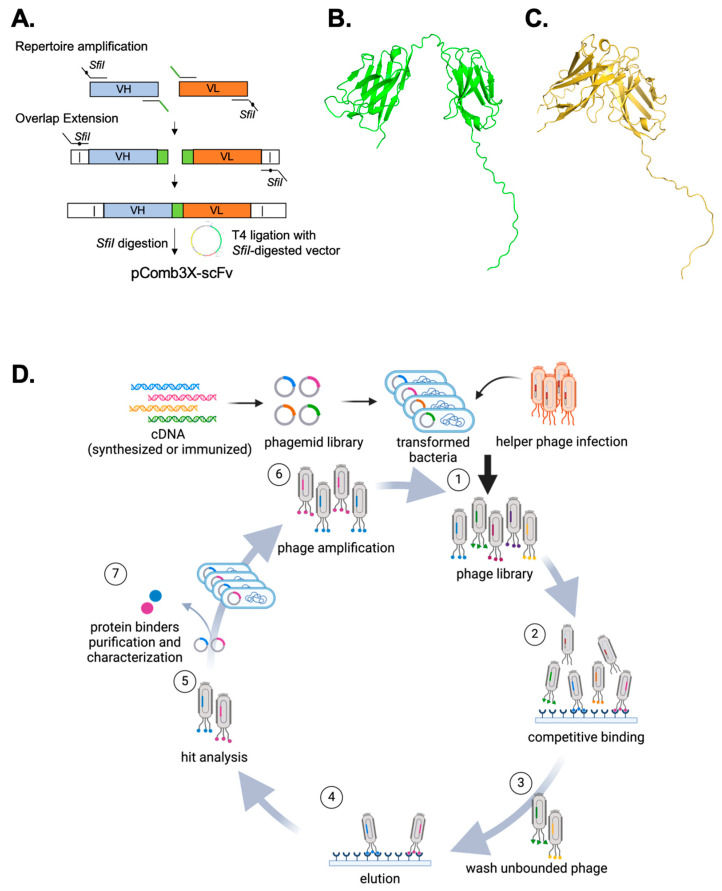
Workflow of scFv library generation and phage display affinity selection. (**A**) Repertoire amplification: variable heavy (V_H_) and variable light (V_L_) regions (including both κ and λ chains) are PCR-amplified from the cDNA of human peripheral blood mononuclear cells (PBMCs) using primers containing *SfiI* restriction sites (indicated by dots). During amplification, short or long overlapping linker sequences (highlighted in green) are introduced upstream of V_H_ and downstream of V_L_. Overlap extension: a subsequent PCR promotes the annealing of V_H_ to V_L_ with either short or long linkers, resulting in the formation of scFv constructs. These constructs are digested with *SfiI* and ligated into the *SfiI*-linearized pComb3XSS vector, creating the scFv phagemid library for subsequent phage display affinity selection toward specific antigens. (**B**,**C**) Structural predictions of scFvs: AlphaFold-predicted structures of scFvs with a short linker (V_H_–S–V_L_) (**B**) and a long linker (V_H_–L–V_L_) (**C**). The V_H_-S-V_L_ is predisposed to form bispecific diabodies, whereas the V_H_–L–V_L_ likely maintains a structure akin to the natural antibody paratope. (**D**) Phage display affinity selection procedure: the scFv phagemid library is introduced into F-pilus-bearing bacteria and subsequently infected with helper phages, resulting in the production of phages that display scFvs. These phages are then subjected to successive rounds of screening to bind the target antigen, as illustrated in steps 1–6. Typically, three to five rounds of affinity selection are necessary to isolate scFvs that bind with high affinity to the target antigen.

This protocol delineates a comprehensive workflow for generating a highly diverse human scFv library from multiple donors. It includes an extensive set of primers for amplifying V_H_ and V_L_ (both κ and λ light chains), ensuring maximal library diversity. Moreover, the protocol describes subsequent phage display affinity selection procedures aimed at identifying scFvs that specifically recognize the target antigen with high binding affinity, utilizing high-throughput monoclonal ELISA (Figure 2). To date, this protocol has effectively created libraries specific to pathogens and identified numerous binders specific to various antigens. The reliability of the results has been established across a wide array of protein types. In summary, this protocol provides a thorough and well-devised procedure for generating scFv libraries, as well as an extensive account of the associated phage display screening process. The technique specified has demonstrated reliability and repeatability, holding promise for diverse applications within molecular biology and therapeutic development.

## 2. Experimental Design

### 2.1. Materials

1. SuperScript^TM^ III First-Strand Synthesis System (Invitrogen, Waltham, MA, USA; Cat. no.: 18080051).
2. Oligo(dT)_12–18_ Primer (Invitrogen, Waltham, MA, USA; Cat. no.: 18418012).
3. Platinum^TM^ Hot Start PCR Master Mix (2×) (Invitrogen, Waltham, MA, USA; Cat. no.: 13000012).
4. UltraPure^TM^ DNase/RNase-Free Distilled Water (Invitrogen, Waltham, MA, USA; Cat. no.: 10977015).
5. Gel loading dye, purple, 6× (New England Biolabs, Ipswich, MA, USA; Cat. no.: B7024S).
6. SeaKem LE agarose (Lonza, Basel, Switzerland; Cat. no.: 50004).
7. Ethyl alcohol, pure (Sigma-Aldrich, St. Louis, MO, USA; Cat. no.: E7023).
8. Sodium acetate, 3 M, pH 5.2, molecular biology grade (Millipore Sigma, Burlington, MA, USA; Cat. no.: 567422).
9. Zymoclean Gel DNA Recovery kit (capped columns) (Zymo Research, Irvine, CA, USA; Cat. no.: D4007/D4008).
10. pComb3XSS plasmid vector (Addgene, Watertown, MA, USA; Cat. no.: 63890).
11. *SfiI* restriction enzyme (New England Biolabs, Ipswich, MA, USA; Cat. no.: R0123S).
12. T4 DNA ligase (New England Biolabs, Ipswich, MA, USA; Cat. no.: M0202S).
13. T4 DNA ligase reaction buffer (New England Biolabs, Ipswich, MA, USA; Cat. no.: B0202S).
14. XL1-Blue Electroporation-competent cells (pUC18 plasmid included) (Agilent, Santa Clara, CA, USA; Cat. no.: 200228).
15. GlycoBlue Co-precipitant (15 mg/mL) (Invitrogen, Waltham, MA, USA; Cat. no.: AM9516).
16. Electroporation cuvettes, 0.2 cm (Invitrogen, Waltham, MA, USA; Cat. no.: P45050).
17. 15 mL Falcon conical centrifuge tube (Fisher scientific, Hampton, NH, USA; Cat. no.: 14-959-53A).
18. 50 mL Falcon high-clarity conical centrifuge tube (Fisher scientific, Hampton, NH, USA; Cat. no.: 14-432-22).
19. SOC medium (Thermo Fisher scientific, Waltham, MA, USA; Cat. no.: 15544034).
20. GeneJET Plasmid miniprep kit (Thermo Fisher scientific, Waltham, MA, USA; Cat. no.: K0502).
21. Falcon 14 mL round-bottom test tubes with cap (Fisher scientific, Hampton, NH, USA; Cat. no.: 14-959-11B).
22. CM13 interference-resistant helper phage (Antibody design labs, San Diego, CA, USA; Cat. no.: PH020L).
23. Nunc MaxiSorp ELISA plate (Thermo Fisher scientific, Waltham, MA, USA; Cat. no.: 442404).
24. Immulon 4HBX ELISA plate (Thermo Fisher scientific, Waltham, MA, USA; Cat. no.: 3855).
25. Glucose (Thermo Fisher scientific, Waltham, MA, USA; Cat. no.: 15023021).
26. Ampicillin sodium salt (Millipore Sigma, Burlington, MA, USA; Cat. no.: A9518).
27. Carbenicillin disodium salt (Millipore Sigma, Burlington, MA, USA; Cat. no.: C3416).
28. Tetracycline (Thermo Fisher scientific, Waltham, MA, USA; Cat. No.: J61714-14).
29. Kanamycin disulfate salt (Millipore Sigma, Burlington, MA, USA; Cat. no.: K1876).
30. LB broth base powder (Thermo Fisher scientific, Waltham, MA, USA; Cat. no.: 12780052).
31. LB agar powder (Thermo Fisher scientific, Waltham, MA, USA; Cat. no.: 2270025).
32. Sulfuric acid (H_2_SO_4_) (Millipore Sigma, Burlington, MA, USA; Cat. no.: 339741).
33. Hydrochloric acid (HCl) (Millipore Sigma, Burlington, MA, USA; Cat. no.: 320331).
34. Sodium chloride (NaCl) (Millipore Sigma, Burlington, MA, USA; Cat. no. 13423).
35. Yeast extract (Millipore Sigma, Burlington, MA, USA; Cat. no.: Y1625).
36. Bacto-tryptone (Thermo Fisher scientific, Waltham, MA, USA; Cat. no.: 211705).
37. A 0.2 μm vacuum filter system (Millipore Sigma, Burlington, MA, USA; Cat. no.: Z358193).
38. Polyethylene glycol (PEG)-6000 (Millipore Sigma, Burlington, MA, USA; Cat. no.: 807491).
39. Tween-20 (Millipore Sigma, Burlington, MA, USA; Cat. no.: P1379).
40. Phosphate-buffered saline (PBS) (Thermo Fisher scientific, Waltham, MA, USA; Cat. no.: 10010023).
41. AEBSF, hydrochloride (Millipore Sigma, Burlington, MA, USA; Cat. no.: 101500)
42. Trypsin (Millipore Sigma, Burlington, MA, USA; Cat. no.: T4799).
43. M13 phage coat protein monoclonal antibody (clone A5B3), HRP (Thermo Fisher scientific, Waltham, MA, USA; Cat. no.: MA5-36125).
44. OptEIA^TM^ TMB substrate reagent set (BD Biosciences, Franklin Lakes, NJ, USA, Cat. no.: 555214).
45. Polycarbonate Erlenmeyer Flask (battled base), 250 mL (Thomas scientific, Chadds Ford Township, PA, USA; Cat. no.: FBC0250S).
46. TG1 phage competent cells (Antibody design labs, San Diego, CA, USA; Cat. no.: PC001).
47. Falcon 96-well, non-treated round-bottom plate (Fisher scientific, Hampton, NH, USA; Cat. no.: 08-772-54).
48. PBMCs isolated from patients’ blood were stored in RNAlater solution at −20 °C.

### 2.2. Equipment

- C1000 Touch Thermal Cycler with Dual 48/48 Fast Reaction Module (Bio-Rad, Hercules, CA, USA; Cat. no.: 1851148).
- Gene Pulser II Electroporation system (Bio-Rad, Hercules, CA, USA; Cat. no.: 165–2110).
- Vac-Man Laboratory vacuum manifold (Promega, Madison, WI, USA; Cat. no.: A7231).
- FilterMax F5 Multi-Mode Microplate reader (Molecular devices, San Jose, CA, USA; Cat. no.: F5).
- Heidolph Titramax 101 Vibrating platform shaker (Marshall scientific, Hampton, NH, USA; Cat. no.: H036130080).
- Thermo MaxQ 6000 Incubated/Refrigerated shaker (Thermo Fisher scientific, Waltham, MA, USA, Cat. no.: SHKE6000-8CE).
- Thermo MaxQ 8000 Incubated/Refrigerated shaker (Thermo Fisher scientific, Waltham, MA, USA; Cat. no.: SHKE8000-8CE).

## 3. Procedure

### 3.1. First Strand cDNA Synthesis (Reverse Transcription, 1 Day)

This protocol begins with the reverse transcription of variable heavy (V_H_) and variable light (V_L_) chain genes from whole blood or peripheral blood mononuclear cells (PBMCs) from human donors. Researchers may choose any RNA extraction kit or protocol that is optimal for the sample type to achieve the desired diversity of the final scFv library. While the quantity of human donor samples used greatly depends on the availability of resources and the specific aims of the research, we extracted total RNA from PBMCs from 19 donors. The extracted total RNA quality needs to be assessed via gel electrophoresis before proceeding to reverse transcription to generate V_H_ and V_L_ cDNA. High-quality, intact RNA should exhibit distinct bands at 4.2 kb and 1.8 kb, corresponding to the 28S and 18S rRNA subunits, in a 2:1 intensity ratio, respectively.

1. Quantify the RNA concentration in the purified RNA. If the RNA concentration is low, the concentration of the RNA is recommended prior to the next steps. Aim for a final concentration of 40 μg of total RNA in 128 μL of ultrapure water. If the concentration exceeds this, dilute the RNA accordingly.
2. To the RNA, add 16 μL of 50 μM oligo(dT)_12–18_ and 16 μL of 10 mM dNTP mix to achieve a final volume of 160 μL. Incubate at 65 °C for 5 min, then place on ice for at least 1 min. Briefly centrifuge to collect any liquid from the cap or side of the tube.
3. In a separate RNase-free microcentrifuge tube, prepare a reverse transcription mix by combining 32 μL of 10× RT buffer, 64 μL of 25 mM MgCl_2_, 32 μL of 100 mM DTT, 16 μL of 40 U/μL RNaseOUT, and 16 μL of 200 U/μL SuperScript III reverse transcriptase for a total of 160 μL.
4. Combine the reverse transcription mix with the RNA/Oligo/dNTP mixture to reach a total volume of 320 μL.
5. Incubate the mixture at 50 °C for 50 min, then at 85 °C for 5 min. Cool on ice for at least 1 min.
6. Add 16 μL of 2 U/μL *E. coli* RNase H and incubate for 20 min at 37 °C. Centrifuge briefly and pool the resultant first-strand cDNAs from different tubes.

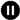
 **PAUSE STEP** The first-strand cDNA may be stored at −20 °C for several weeks. For extended storage (several months to years), add 0.1 volume (33.6 μL) of 3 M sodium acetate (pH 5.2) and 2.2 volume (739.2 μL) of EtOH, vortex to mix well, and store at −80 °C.

### 3.2. scFv Construction (6–10 Days)

#### 3.2.1. V Gene Amplification (3–7 Days)

The initial stage in scFv generation involves the amplification of the variable region (Figure 2A). The human genome contains three loci associated with antibodies: one for the heavy chain and two for the light chains, kappa (κ) and lambda (λ). The heavy chain consists of the variability (V), diversity (D), joining (J), and constant (C) segments. In contrast, both κ and λ light chains comprise only V and J segments (Table 1). The V, D, and J segments are further divided into various families. To encompass the entire immune repertoire, we designed 348 primer combinations that target all V families (forward primers) and J families (reverse primers). These primers, based on Rader et al.’s work and subsequently modified in accordance with Barbas et al. and the V base database [29,30], have been tailored to include sequences for both short and long linkers. The sequences of these primers are detailed in Appendix A.

The scFv molecules can be assembled in two configurations: V_H_-linker-V_L_ or V_L_-linker-V_H_. This protocol employs the V_H_-linker-V_L_ orientation, which has been demonstrated to exhibit higher binding affinity for the target antigen due to a more exposed V_H_ CDR3 [8,32]. V_H_ and V_L_ regions are connected by either a short glycine and serine-rich flexible linker (GGSSRSS) or a long linker (SSGGGGSGGGGGGSSRSS) [30]. ScFvs with the short linker are predisposed to forming bispecific diabodies (Figure 2B), while those with the long linker are more likely to mimic the natural elbow structure of the paratope, as predicted by AlphaFold (Figure 2C) [32,33,34]. Empirically, we have determined that long linker constructs are amplified more efficiently when positioned downstream of the V_H_ rather than upstream of V_L_. Consequently, we produce four distinct pools of PCR products: V_H_ with a short linker (V_H_-S), V_H_ with a long linker (V_H_-L), kappa light chain (V_κ_), and lambda light chain (V_λ_), each derived from the corresponding antibody fragment isolated from the reactions. To mitigate nonspecific amplification and primer dimer formation during PCR, we utilize a Touch Down PCR protocol in lieu of a conventional PCR approach. The resulting amplified V region genes should be approximately 400 bp in size.

1. Prepare 348 unique PCR master mixes, with each PCR primer combination, and conduct Touch Down PCR as follows (Table 2):

2. Verify the PCR amplification success on a 1.5% agarose gel. Combine 3 μL of the PCR product with 2 μL of 6× DNA gel loading dye and perform gel electrophoresis at 135 V for 20 min.
3. Proceed with ethanol precipitation for PCR products that show successful amplification.

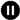
 **PAUSE STEP** The PCR product can be stored at 4 °C for up to 7 days if not proceeding immediately.

#### 3.2.2. Ethanol (EtOH) Precipitation of Pooled PCR Reactions (1 Day)

EtOH precipitation is employed to purify PCR products by removing enzymes and salts. Note that dNTPs and primers might co-precipitate, which could interfere with the Abs_260nm_ and subsequent DNA quantification by UV spectrophotometry. To minimize this, replace sodium acetate with ammonium acetate in the protocol below.

1. Combine the PCR products for each V region into a separate 15 mL Falcon tube, ensuring you prepare four tubes for the four V regions. Mix thoroughly, then aliquot 330 μL into individual 1.5 mL microcentrifuge tubes.

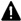
 **CRITICAL STEP** It is essential to ensure a consistent mix in the 15 mL Falcon tubes before aliquoting, to maintain diversity and homogeneity, as some DNA might be lost during the EtOH precipitation process.

2. Add 0.1 volume (33 μL) of 3 M sodium acetate to each tube, followed by 3 volumes (1089 μL) of 100% EtOH. Incubate at −20 °C overnight to precipitate the DNA.
3. Centrifuge at 16,000× *g* for 10 min at room temperature (RT) and aspirate most of the EtOH without disturbing the pellet.
4. Wash the DNA pellet with 1 mL of ice-cold 70% EtOH (in ultrapure water) and centrifuge at 16,000× g for 2 min at RT. Carefully aspirate the ethanol without disturbing the pellet. Repeat this wash.

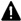
 **CRITICAL STEP** Ice-cold 70% EtOH is recommended to prevent DNA loss, as DNA may dissolve in the aqueous portion of RT 70% EtOH.

5. After the second wash, centrifuge at 16,000× g for 2 min at RT and remove all the EtOH.
6. Air-dry the pellet for 15 min to an hour with the tube lid open to ensure it is completely dry.
7. Resuspend the DNA pellet from each tube in 30 μL ultrapure water (preheated to 55 °C), and pool the resuspensions into four 4 tubes (one for each V fragment).
8. Run 25 μL of each resuspended V fragment DNA on a 1.5% agarose gel at 100 V for 40 min, using multiple lanes as needed to fit all samples.

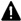
 **CRITICAL STEP** Employ low voltage for gel electrophoresis to prevent heat-induced DNA damage.

9. Extract DNA from the gel using a Zymoclean Gel DNA Recovery kit and elute in water heated to 55 °C to maximize yield. Determine DNA using a spectrophotometer.

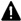
 **CRITICAL STEP** This step is crucial to diminish primer interference from the first PCR in subsequent amplifications.


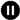
 **PAUSE STEP** Store the purified DNA at −20 °C for long-term storage.

#### 3.2.3. Secondary Overlapping Extension (SOE) PCR (2 Days)

Following the amplification of V regions, V_H_-S and V_H_-L amplicons are designed to contain sequences that overlap with V_κ_ and V_λ_ for fusion. After fusion, these constructs are further amplified via SOE PCR, resulting in four different scFv constructs: V_H_-S-Vκ, V_H_-S-V_λ_, V_H_-L-V_κ_, and V_H_-L-V_λ_. A minimum of 15 μg of each construct is necessary for subsequent experiments. Typically, conducting 12 SOE PCR reactions for each scFv combination (totaling 48 reactions) yields a sufficient DNA concentration for the next step.

1. Use 50 ng of each V_H_ and V_L_, along with 5 μL of primer mix, for the reactions. We recommend performing 12 SOE reactions for each construct to ensure an adequate quantity of DNA is produced. Execute the PCR according to the protocol outlined in Table 3.

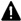
 **CRITICAL STEP** Performing 20 cycles prior to an infinite hold at 12 °C is crucial. Fewer than 20 cycles may result in indistinct scFv bands, while more than 20 cycles can impair polymerase activity.

2. Assess the SOE PCR success by electrophoresing 3 μL from each of the 48 tubes on a 1.5% agarose gel.
3. Pool the remaining SOE PCR products into four separate microcentrifuge tubes.
4. Repeat DNA EtOH precipitation as described in Section 3.2.2, “EtOH precipitation of pooled PCR reactions.” Combine approximately 360 μL of the reaction mixture from 12 PCR reactions per construct, adding 0.1 volume (30 μL) of 3 M sodium acetate, followed by 3 volumes (1080 μL) of 100% EtOH.
5. Precipitate DNA overnight at −20 °C.
6. The following day, wash the precipitated DNA as outlined in Section 3.2.2, “EtOH precipitation of pooled PCR reactions.”
7. Utilize the required amount for digestion as detailed in Section 3.3, “Cloning scFv constructs to pComb3XSS by *SfiI* digestion and T4 DNA ligase reaction,” or store the remainder of the DNA.

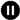
 **PAUSE STEP** The precipitated DNA can be stored at −20 °C for several weeks.

### 3.3. SfiI Digestion of pComb3XSS and scFv Constructs (2 Days)

The amplified scFv constructs include *SfiI* digestion sites for sticky end formation compatible with the pComb3XSS vector for cloning. 500 ng of each digested scFv construct is needed for library generation.

1. Digest 50 μg of the pComb3XSS vector with 10 μL of *SfiI* enzyme (4 units/μg of DNA), bringing the total volume to 250 μL with water (50 μL for every 10 μg of DNA).
2. Digest 15 μg of the scFv construct precipitated from the prior step with 3 μL of *SfiI* enzyme, adjusting the final volume to 150 μL with water (50 μL for every 5 μg of DNA).
3. Incubate the digestion reactions at 37 °C overnight.
4. **OPTIONAL** Despite the manufacturer’s recommendations for a 60 °C reaction temperature, 37 °C may be used for stable and prolonged digestion.
5. Confirm complete digestion by comparing the digested fragments with the undigested vector on a 1.5% agarose gel.
6. Use a Zymoclean Gel DNA Recovery kit to gel-extract the fully digested pComb3Xss (3379 bp), the stuffer fragment (1673 bp—removed from the pComb3XSS), and scFv constructs (700–800 bp). Elute with water preheated to 55 °C to maximize yield.

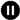
 **PAUSE STEP** Store the eluted DNA at −20 °C for several weeks.

### 3.4. Test Library Generation (2 Days)

To ensure the generation of a library with a size greater than 1 × 10^7^, a test library should be constructed to determine the necessary number of ligation reactions. T4 DNA ligase is used to ligate the digested scFv into the pComb3XSS vector. While alternative methods like Golden Gate assembly may serve the same purpose, our protocol specifically details the use of the traditional T4 DNA ligase reaction for its broad applicability. The test library is produced by direct electroporation of the ligation product into XL1-Blue electrocompetent cells. While this protocol specifically utilizes XL1-Blue, any bacterial strain that harbors the F-factor, such as TG1 or XL1-Blue, is suitable for generating libraries and subsequent affinity selection due to their ability to support filamentous phage infection.

#### 3.4.1. Single T4 DNA Ligase Reaction of scFv into pComb3xSS (Adapted from [14], 1 Day)

1. Prepare four ligation reactions as follows, which include three controls and the scFv ligation reactions for each construct:
  - Control #1: without ligase;
  - Control #2: without insert;
  - Control #3: with stuffer insert;
  - Four separate scFv ligations for each scFv construct
  - The composition and volumes for these reactions are detailed in the table provided (Table 4).
2. Carry out the T4 DNA ligase reaction according to the protocol specified in the subsequent table (Table 4).

3. On the following day, combine 1 μL from each of the four scFv construct ligation reactions (totaling 20 μL) into a new microcentrifuge tube, resulting in a total volume of 4 μL. This mixture will be electroporated into 50 μL of XL1-Blue competent cells.
4. **OPTIONAL STEP** For quality control of individual scFv constructs, heat-shock TOP10 competent cells with 1 μL of each ligation product (four scFvs separately) and perform a dilution series inoculation on LB agar plates supplemented with ampicillin to assess diversity size.

#### 3.4.2. Test Library Electroporation (1 Day)

1. In a new 1.5 mL microcentrifuge tube, combine 1 μL from each of the four scFv ligation products for electroporation into 50 μL of XL1-Blue competent cells.
2. Pre-warm SOC media to 37 °C and prepare 2 mL of pre-warmed SOC media in 15 mL Falcon tubes.
3. Thaw 250 μL of XL1-Blue competent cells, allocating 50 μL for each of the five electroporation samples. These include three negative controls (no ligase, no insert control, and stuffer insert) and the combined scFv sample (4 μL total), along with a pUC18 electroporation control.
4. Aliquot 50 μL of competent cells into each pre-cooled microcentrifuge tube: mix 50 μL of competent cells with 4 μL of the ligation product for each control and scFv, along with 1 μL of 0.1 ng/μL pUC18 plasmid as a positive control. Mix gently by pipetting and transfer into 0.2 cm gap electroporation cuvettes, avoiding bubble formation.
5. Set the Gene Pulser II apparatus to 25 μF capacitance, 2.5 kV, and 200 Ohm.
6. Tap the cuvette gently to remove bubbles and ensure all cells settle at the bottom, free from bubbles.

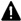
 **CRITICAL STEP** The pulse should result in 12.5 kV/cm with a time constant of 4–5 milliseconds.

7. Immediately add 950 μL of pre-warmed SOC to the electroporated cuvette, and transfer the mixture to the prepared 15 mL Falcon tubes containing 2 mL of SOC, making a total volume of 3 mL. Incubate at 37 °C with shaking at 225 rpm for 1 h. Meanwhile, pre-warm four LB agar plates supplemented with ampicillin to 37 °C.

**OPTIONAL** Given carbenicillin’s enhanced stability compared to ampicillin, it can be used as a preferable alternative at working concentrations ranging from 50 to 100 µg/mL.

8. After 1 h of recovery, take 10 μL from the transformed cells and from the pUC18-transformed cells to estimate the library size. Perform tenfold serial dilutions up to 10^−8^ in a 96-well plate. Drop 5 μL from each dilution onto a pre-warmed LB agar plate with ampicillin using a multichannel pipette or a 96-pin pronger. Plate the three ligation controls (no ligase, no insert, and stuffer insert) at a 1:100 dilution on separate LB agar plates.
9. Incubate all plates at 37 °C overnight (approximately 16 h).
10. The following day, calculate the library size from the test library using the formula:

$$\;Estimated\;library\;size\;from\;single\;reaction=\;\;Number\;of\;colonies\;on\;plate\times dilution\;factor\times \frac{volume\;of\;ligation\;product\;(=20\;\mu L)}{volume\;of\;electroporation\;product\;(=4\;\mu L)}$$

### 3.5. Direct (Large-Scale) Library Generation (3 Days)

#### 3.5.1. Large-Scale Ligation (1 Day)

Calculate the number of ligation reactions needed to achieve a library size of 10^7^ or 10^8^, based on test library results. For direct ligation, it is necessary to precipitate the ligated DNA to minimize salt interference, which can decrease electroporation efficiency and cell viability. For example, if a single ligation yields a library size of 5 × 10^6^, then four ligation reactions are needed to surpass 1 × 10^7^. The following steps apply to a single reaction; adjust quantities accordingly for the desired library size. After EtOH precipitation, combine all scFv constructs separately into four tubes. We recommend performing an equal number of ligation reactions for each scFv construct to ensure a balanced representation of each in the pooled ligation product.

1. Conduct multiple ligation reactions to reach the target library > 10^7^ size.
2. Follow the T4 DNA ligase reaction protocol as outlined in Section 3.4.1, “Single T4 DNA ligase reaction of scFv into pComb3XSS.”

#### 3.5.2. EtOH Precipitation of the Ligated scFv Phagemid Library (1 Day)

1. Pool the ligation reactions up to a maximum of 330 μL per tube. Add 0.1 volume of 3 M sodium acetate and 3 volumes of EtOH, as described in Section 3.2.2, “EtOH precipitation of pooled PCR reactions.”

**OPTIONAL** For smaller numbers of ligation reactions or low DNA concentrations, add a co-precipitant like GlycoBlue or Glycogen at a 1:300 dilution during EtOH precipitation to enhance precipitation efficiency.

2. The next day, centrifuge the precipitated DNA and wash the pellet following the steps in Section 3.2.2, “EtOH precipitation of pooled PCR reactions.” Resuspend the pellet in a total of 200 μL of ultrapure water warmed to 55 °C.

#### 3.5.3. Electroporation into XL1-Blue Competent Cells (1 Day)

Electroporate 250 ng of the precipitated ligation product into 50 μL of XL1-Blue competent cells. Using an excessive amount of DNA relative to the number of cells might result in the introduction of multiple DNA molecules into a single cell. Note that spectrophotometer readings may be inaccurate due to salt. The theoretical yield of precipitated DNA is calculated as follows: if 20 ligation reactions are precipitated, with each digested vector (3379 bp) yielding 171 ng of ligated scFv-carrying phagemid, the total yield will be 3420 ng in 200 μL water (17.1 ng/μL). Therefore, 14.6 μL of the precipitated DNA (equivalent to 250 ng) is needed for each 50 μL aliquot of competent cells. In the actual library generation, the three negative ligation controls are unnecessary if using the same batch of digested vectors as in the test library.

1. Pre-cool the electroporation adaptor, a 15 mL Falcon tube, a microcentrifuge tube, and electroporation cuvettes (0.2 cm) on ice.
2. Pre-warm 50 mL Falcon tubes containing 3 mL of SOC medium and 5 mL of SOC medium to 37 °C.
3. Prepare the required volume of 50 μL competent cell mixed with 14.6 μL DNA (based on calculations to reach the desired library size), mixing slowly for thorough integration.
4. Prepare a control mixture with pUC18 and competent cells in a separate microcentrifuge tube.
5. Aliquot the scFv master mix into the electroporation cuvettes, ensuring the absence of air bubbles. Wipe the cuvettes’ metal surfaces to remove moisture.
6. Repeat the electroporation process as in Section 3.4.2, “Test library electroporation.”
7. Immediately add 935 μL of warmed SOC media to each cuvette and transfer the bacteria to the pre-warmed 50 mL Falcon tubes. Ensure each tube does not exceed 10 mL of total volume for adequate aeration.

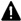
 **CRITICAL STEP** The volume of bacteria in the Falcon tube should not exceed 20% of the tube’s volume to aerate bacteria for growth. Thus, add only up to 7 electroporated bacteria (each 1 mL) to each 50 mL Falcon tube containing 3 mL of SOC media.

8. Shake the electroporated bacteria at 37 °C and 225 rpm for 1 h for recovery.
9. For library size calculation, dilute 100 μL from the Falcon tube for serial dilutions to 10^−8^, then plate 5 μL from each dilution. Store the remaining dilutions at 4 °C.
10. Spin down the rest of the bacteria and resuspend in a total of 4 mL. Incubate 1 mL onto each of several 245 mm square LB agar plates (with 2% *w*/*v* glucose and 50 μg/mL ampicillin).
11. Incubate the plates overnight at 37 °C.
12. Scrape the bacteria from the plates using 4 mL of LB media, rinsing with an additional 2 mL of LB. Be gentle to avoid scraping the agar.
13. Add glycerol to a final concentration of 20%. Dilute 100 μL of the scraped bacteria in 900 μL LB and measure the OD at 600 nm (OD_600_). An OD_600_ corresponds to 1 × 10^8^ bacteria per mL.

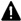
 **CRITICAL STEP** Dilute the bacteria before measuring OD_600_ to avoid exceeding the spectrophotometer’s detection limit.

14. Aliquot the bacteria to achieve 5 × 10^9^ bacteria per cryovial for storage at −80 °C.

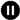
 **PAUSE STEP** The scFv bacteria library can be stored at −80 °C for years.

15. **OPTIONAL STEP** Adjust the number of bacteria per vial based on library diversity. A higher number of bacteria based on library diversity can be used so that each library stock contains 48 or more copies per scFv repertoire [35]. For example, if the diversity size is 8 × 10^8^, 4 × 10^10^ bacteria can be aliquoted to each cryovial so that each stock contains 50 copies of each scFv in the library. However, avoid exceeding 1 × 10^12^ bacteria per vial to prevent spontaneous phage precipitation.

### 3.6. Library Quality Assessment (2–3 Days)

Quality control (QC) of the final library can include methods such as colony PCR, Sanger sequencing, *SfiI* digestion, or *BstNI* scFv fingerprinting. We recommend Sanger sequencing for identifying scFv sequences and assessing library diversity.

1. Thaw a vial of the library stock and prepare a serial dilution series to 10^−8^.
2. Inoculate 100 μL of each dilution onto LB agar plates containing 2% *w*/*v* glucose, 50 μg/mL ampicillin.

**OPTIONAL** When the cultivating library is XL1-Blue cells, it is recommended to add tetracycline to the culture medium at a concentration of 50 µg/mL.

3. Incubate the plates at 37 °C overnight to allow colony growth.
4. Pick individual colonies and inoculate them into 3 mL of LB media supplemented with ampicillin, using Falcon 14 mL round-bottom tubes.
5. Incubate these cultures at 37 °C overnight to promote further colony growth.
6. Perform a miniprep using the GeneJET Miniprep kit to extract bacterial plasmid.

**OPTIONAL** For higher throughput, consider using a vacuum manifold during the miniprep process.

7. Sequence the extracted DNA using Sanger sequencing with the sequencing primers provided in Appendix A.

### 3.7. Phage Display Affinity Selection (Variable Time Depending on Enrichment, Usually 14–20 Days)

#### 3.7.1. Optimization of Antigen Coating Condition on Different ELISA Plates (1–2 Days)

Optimizing ELISA plate coating concentration is crucial to conserve protein and maximize affinity selection efficiency. Different proteins adhere differently to various types of ELISA plates. For instance, glycoproteins often coat effectively on Nunc MaxiSorp or Immulon 4HBX plates, while lipoproteins may prefer other types [36]. Consult the manufacturer’s catalog to choose the optimal ELISA plate for coating your target protein. Additionally, consider the antigen capacity of the plates, but note that this varies with each protein. When selecting antigens for negative selection, consider relevant factors; for instance, use Fc-tagged target proteins, including Fc for negative selection, or BSA if no similar protein is available.

1. Select appropriate ELISA plates for optimal coating of the target protein.
2. Set various antigen coating concentrations from 1–5 µg/mL based on the manufacturer’s guidelines. Target and control antigen proteins should be in similar forms.
3. Coat the ELISA plates, 100 µL per well, with triplicates of both target and control antigens in PBS (or an appropriate buffer for the antigens) and incubate overnight at 4 °C with gentle shaking.
4. Wash the plates three times with PBS-T (0.05% Tween-20 in PBS).
5. Block the plates using 200 μL of 5% milk in PBS-T, incubating for 1 h at RT with gentle shaking.
6. Wash the plates three times with PBS-T.
7. Add 100 μL of HRP-conjugated antibody specific to the target or tag of the target protein and control antigens. Follow the manufacturer’s guidelines and incubate for 1 h at RT with gentle shaking.

**OPTIONAL** In the absence of an HRP-conjugated antibody specific to the target and control antigens, an indirect ELISA approach can be employed. This involves using a primary antibody that specifically recognizes the target and control antigens, followed by detection with an HRP-conjugated secondary antibody.

8. Wash the plates three times with PBS-T.
9. Develop the reaction with 100 μL of TMB substrate for 5 to 20 min, then stop the reaction with 50 μL of 1 M H_2_SO_4_ (or 1 N HCl). Measure the signal intensity at 450 nm.
10. Plot a titration curve of OD_450nm_ against different antigen coating concentrations to find the optimal concentration for affinity selection.

#### 3.7.2. Induction of Phages Carrying scFv and In Vitro Selection (Multiple Rounds of 3 Days)

This phage display protocol is adapted and modified from Pardon et al. and Lee et al. [35,37], starting with the scFv library stock constructed in Section 3.5.3, “Electroporation into XL1-Blue competent cells.”

1. Inoculate the library stock into 60 mL of 2xTY-G supplied with 50 µg/mL ampicillin (and 50 µg/mL tetracycline for XL1-Blue cells). Rinse the tube to collect all bacteria.
2. Reamplify the bacteria library culture at 37 °C and 200 rpm until OD_600_ reaches 0.5.
3. Transfer 10 mL of the exponential-phase bacteria to a 50 mL Falcon tube and infect with 4 × 10^10^ cfu of VCSM13 helper phage. Incubate for 30 min at 37 °C without shaking.

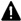
 **CRITICAL STEP** Use filtered pipette tips and disinfect the tips before disposal to prevent phage contamination. Used pipette tips should be discarded in a biohazard bag for either dispensing or autoclaving later.

4. Centrifuge the cells at 4000× *g* for 10 min at RT. Discard the supernatant, and briefly place the tube on a paper towel.
5. Resuspend in 60 mL of 2xTY-I and transfer to a disposable 250 mL baffled flask. Incubate overnight at 37 °C and 200 rpm to induce the phages carrying scFv.
6. The next day, start growing naïve TG1 (not carrying the scFv gene) in 10 mL LB media in a 50 mL Falcon tube. Grow until OD_600_ reaches 0.5 to 0.6, then keep in ice.

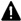
 **CRITICAL STEP** TG1 cells should be first cultured in M9 medium to ensure the presence of F-factor.

7. Centrifuge the induced 50 mL culture at 4000× *g* for 30 min at 4 °C.
8. Transfer 40 mL of supernatant to a new 50 mL Falcon tube, then add 10 mL of PEG solution. Mix well by inverting the tube 5 times.
9. Incubate the mixture in ice for an hour to precipitate the phages.
10. Spin down at 4000× *g* for 30 min at 4 °C. Discard the supernatant and place the tube upside down on a paper towel briefly to remove residual supernatant.
11. Resuspend the precipitated phage pellet using 1 mL of ice-cold PBS, then transfer the resuspension to a new ice-cold microcentrifuge tube.
12. Centrifuge at 20,000× *g* for 1 min at 4 °C to spin down residual bacteria.
13. Carefully transfer only the supernatant to a new ice-cold microcentrifuge tube.
14. Add 250 μL of ice-cold PEG solution and pipet up and down several times to mix well.
15. Incubate on ice for 10 min.
16. Spin down the phages at 14,000× *g* for 15 min at 4 °C. Discard the supernatant and resuspend the pellet using 1 mL of ice-cold PBS.
17. Centrifuge at 14,000× *g* for 1 min at 4 °C to completely remove residual bacteria.
18. Transfer the supernatant to a new ice-cold microcentrifuge tube.
19. To a round bottom 96-well plate, add 90 μL of PBS to a row of 12 wells. Use this PBS to make 12 tenfold serial dilutions of the precipitated phage, beginning with 10 μL of precipitated phage. Serial dilution can be alternatively performed using a multichannel pipettor if preferred. In a separate row, add 90 μL of naïve TG1 (at OD_600_ = 0.5~0.6) and 10 μL from each serial dilution to achieve a final volume of 100 μL. Incubate the plate at 37 °C for 30 min without shaking.
20. Carefully pipette 5 μL drops of the infected TG1 onto LB agar plates containing 2% glucose and ampicillin. As a control, also pipette 5 μL of naïve TG1 (uninfected by phages). Allow the drops to air-dry and incubate the plates at 37 °C overnight. Concurrently, inoculate a naïve TG1 into 5 mL of LB media (without antibiotics) in a 50 mL Falcon tube for overnight growth.

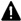
 **CRITICAL STEP** When culturing the naïve TG1 cells, also inoculate TG1 into two tubes containing LB with ampicillin or kanamycin to check for potential contamination by either the phage, helper phage, or both. TG1 contaminated with the induced phage will grow in ampicillin-supplemented media, and TG1 contaminated with helper phage will grow in kanamycin-supplemented media.

21. For antigen coating on ELISA plates, use the previously optimized concentration (as per Section 3.7.1, “Optimization of antigen coating condition on different ELISA plates.” Coat four wells with the control antigen and four non-adjacent wells with the target antigen, using 100 μL for each well. Seal the plate with impermeable film and incubate overnight at 4 °C.
22. The next day, calculate the colony-forming units (cfu) of the precipitated phage using the following formula:

$$\frac{cfu}{\mathrm{mL}}=number\;of\;colonies\;on\;plate\times dilution\;factor\times \frac{total\;volume\;(=1000\;\mu L)}{plated\;volume\;(=5\;\mu L)}$$

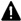
 **CRITICAL STEP** The cfu/mL should be higher than 1 × 10^12^ cfu/mL, which would be at least a hundred times greater than the initial bacterial count. If it is lower, repeat the phage production process.


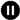
 **PAUSE STEP** Store the precipitated phages at 4 °C for weeks. For long-term storage, add glycerol to achieve a final concentration of approximately 40%. The phage in glycerol can then be stored at −80 °C for several months to years.

23. Inoculate naïve TG1 into 10 mL of LB media in a 50 mL Falcon tube. Grow at 37 °C and 220 rpm until it reaches OD_600_ = 0.5. Then, move the Falcon tube into an ice bucket until it is ready for infection with the eluted phages.
24. Discard the antigen-coating solution from the ELISA plates and wash three times with PBS-T. Block the plates by adding 200 μL of 5% milk in PBS-T to each well, and incubate at RT for 1 h with gentle shaking.
25. During the 1 h blocking, pre-block the phages by adding 5 × 10^11^ cfu of the precipitated phages to 500 μL of 5% milk in PBS-T (1 × 10^11^ per well).

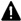
 **CRITICAL STEP** The actual required phage volume is 4 × 10^11^ cfu in 400 μL, but we recommend preparing 5 × 10^11^ cfu in 500 μL to compensate for potential transfer loss. The pre-blocking should be performed for at least 20 min or until the 1 h ELISA plate blocking is complete, with gentle shaking at RT.

26. After blocking the ELISA plates, wash them three times with PBS-T. Transfer 100 μL of the pre-blocked phages to the wells coated with control antigen for negative selection.

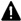
 **CRITICAL STEP** To prevent the target-antigen-coated wells from drying out, add 100 μL of the blocking solution to the wells coated with the target antigen.

27. Agitate the plates gently at 700 rpm on a shaking platform for 2 h.
28. After incubation, remove the blocking solution from wells that have been coated with the target antigen, then transfer the phages from the wells coated with the control antigen to those coated with the target antigen.

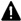
 **CRITICAL STEP** To prevent the controlled-antigen-coated wells from drying out, add 100 μL of the blocking solution to the wells initially coated with the control antigen.

29. Agitate again at 700 rpm for 2 h.
30. After agitation, remove the liquid from all eight wells (four coated with control antigen and four with target antigen). Wash all wells 15 times with 200 μL of PBS-T, followed by a single wash with 200 μL of PBS. Add 50 μL of trypsin (0.25 mg/mL in PBS) to all eight wells and agitate at 700 rpm for 30 min at RT. The addition of trypsin facilitates the dissociation of phages from the target antigen by cleaving the digestion sites between the phage coat protein and the displayed scFv. Phages eluted from both control and target antigens should not be discarded, as they are used in the following steps for enrichment analysis.

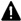
 **CRITICAL STEP** Liquid waste containing phage particles must be treated with a disinfectant before being disposed of at a liquid handling station equipped for regular disinfection.

31. Prepare two microcentrifuge tubes, each containing 10 μL of AEBSF. Transfer the trypsin-eluted phages from the four wells initially coated with control antigen into the first tube, and those from the target antigen wells into the second tube.
32. In a new 50 mL Falcon tube, add 3 mL of exponential-phase naïve TG1 (from step 23). Infect this culture with 50 μL of the eluted phage from the wells initially coated with target antigen. Incubate the mixture at 37 °C without shaking for 30 min. Then, add 7 mL of LB supplemented with ampicillin and glucose to achieve a final concentration of 50 μg/mL ampicillin and 2% glucose in 10 mL. Incubate the 10 mL mixture overnight at 37 °C and 180 rpm.
33. Perform tenfold serial dilutions of trypsin with AEBSF mixtures from control and target Ag, referring to Section 3.7.3, “Enrichment analysis.” If the enrichment factor is less than 1000 (from the first method) but still increasing (from the second method), proceed to the next round of affinity selection.
34. The next day, transfer 500 μL of the overnight-grown 10 mL culture into 50 mL of 2xTY-G (2% glucose, ampicillin) in a baffled disposable flask and incubate at 37 °C, 200 rpm.

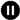
 **PAUSE STEP** Aliquot the remaining 10 mL of culture into cryovials, dispensing 1 mL into each vial. Then, add 666 μL of 50% glycerol to each vial to make the final 20% glycerol, resulting in a total volume of 1.666 mL per cryovial. These can be stored at −80 °C for future single colony picking or for restarting the affinity selection process in case of error.

#### 3.7.3. Enrichment Analysis (1 Day)

Typically, the affinity selection step in phage display requires 3 to 5 rounds, depending on the behavior of the target antigens. However, excessive rounds of affinity selection increase the chance of clonal focusing; therefore, enrichment analysis is recommended to decide when to conclude the selection process. Most antigens require only up to three rounds. Enrichment analysis, a crucial quality control step, assesses the proportion of antigen-specific populations of scFv after each affinity selection round. This protocol describes two methods for evaluating the enrichment of target antigen-specific scFv-carrying phages based on their titers against control and target antigens. Method 1 compares enrichment factors by comparing phage titers against target Ag and control Ag, and Method 2 calculates the ratio of output phages to input phages after each round. Both methods can be used to quantify affinity selection efficiency and the development of target-specific scFvs.

**Method 1: Enrichment factor calculation**

1. Prewarm the LB agar plate (2% *w*/*v* glucose and 50 μg/mL ampicillin) to 37 °C.
2. In a 96-well plate, add 90 μL of PBS to two nonadjacent rows of 12 wells each, making 24 wells in total. Use this to prepare 12 tenfold serial dilutions, starting with 10 μL of the trypsin + AEBSF mixture for both control and target antigens.
3. In two separate rows of 12 wells each, add 90 μL of exponential-phase naïve TG1 (from Section 3.7.2, “Induction of phages carrying scFv and in vitro selection,” step 23).
4. Add 10 μL of each serial dilution to the 90 μL of naïve TG1.
5. Incubate the plate at 37 °C for 30 min without shaking.
6. Using a multichannel pipet, inoculate 5 μL from each well of phage-infected TG1 onto multiple LB agar plates (2% glucose and ampicillin). Use three plates each for the control and target antigens to calculate the average values, considering the sticky nature of phages. Include a control spot of 5 μL of naïve TG1 not infected with phages as a sentinel against contamination by phage.
7. Incubate the plates overnight at 37 °C.
8. The next day, count the average number of colonies from control and target antigens. Calculate the enrichment factor as follows:

$$Enrichment\;factor=\frac{colonies\;observed\;from\;target\;Ag}{colonies\;observed\;from\;control\;Ag}$$

9. An enrichment factor exceeding 1000 is indicative of successful enrichment.

**Method 2: Output/input calculation**

1. Determine cfu from the LB agar plates with drops of TG1 infected with phages eluted from wells coated with the target antigen. For example, if there are three average colonies at the 2nd dilution point (this is 10^−3^ dilution, making the colony number 3 × 10^3^), cfu is $\frac{3\;\times \;10^{3}}{0.005}=6\times 10^{5}/mL$
2. Given that 4 × 10^11^ cfu in 400 μL was used for input (equivalent to 1 × 10^12^/^mL^), calculate the $\frac{output}{input}$ ratio as $\frac{6\;\times \;10^{5}}{1\;\times \;10^{12}}$.
3. This ratio should steadily increase, then plateau or decrease.

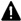
 **CRITICAL STEP** The optimal round to conclude affinity selection and proceed to monoclonal ELISA is the last round before the plateau or decrease in both methods.

#### 3.7.4. Polyclonal ELISA (2 Days)

1. Coat the ELISA plate with control and target antigens at the previously optimized concentration, using 100 μL per well for triplicates. Adjust based on the number of time points being tested in the polyclonal ELISA.
2. Incubate the plate overnight at 4 °C.
3. Wash the plate three times with PBS-T, then block using 200 μL of 5% milk in PBS-T per well for 1 h at RT with gentle shaking.
4. During the blocking period, add 1 × 10^11^ cfu of phages (based on cfu calculation from Enrichment Analysis Method 2) before affinity selection and after each selection round to 5% milk in PBS-T, preparing 100 μL for each time point.
5. After blocking, wash the plates three times with PBS-T and transfer 100 μL of phage in milk to each well.
6. Incubate for 1 h at RT with gentle shaking.
7. Wash the plate three times with PBS-T, add 100 μL of HRP-conjugated anti-M13 coat protein antibody according to the manufacturer’s guidelines, and incubate for 1 h at RT with gentle shaking.
8. Wash the plate three times with PBS-T, then add 100 μL of TMB substrate.
9. Develop the color reaction, then add 50 μL of 1 M H_2_SO_4_ to stop it. Quantify the optical density at 450 nm.
10. Draw a titration curve for OD_450_. If the OD_450_ value plateaus or decreases, affinity selection should be halted at that round. The bacterial stock from the round immediately preceding the plateau or decrease can be used for single colony picking.

### 3.8. Screening for Hits by Monoclonal ELISA (4 Days)

1. Review the results of enrichment analyses (output/input calculation, enrichment factor analysis, and polyclonal ELISA). A plateau or decrease in output/input calculations and polyclonal ELISA, along with an enrichment factor over 1000, indicate it is time to stop affinity selection. For instance, if these criteria are met by the product of Round 3, proceed to pick single colonies from Round 3.
2. Thaw a vial from the −80 °C bacteria stock and prepare a serial dilution series from 10^−1^ to 10^−8^. Inoculate each dilution onto LB agar plates (containing 2% glucose and 50 μg/mL ampicillin) to determine the optimal dilution for picking single colonies. Avoid too many or too few colonies on the plates. Incubate the plates overnight at 37 °C.
3. The next day, visually assess the plates to determine which dilution yields a manageable number of colonies for picking.
4. Plate multiple (10 to 15) LB agar plates (2% glucose and 50 μg/mL ampicillin). Ensure even spreading and drying of the plates to prevent clumping of colonies.
5. Prepare 6 to 12 round-bottom 96-well plates, adding 200 μL of 2xTY-G (2% glucose, 50 μg/mL ampicillin) to each well.
6. Using a sterile P200 pipette tip, pick single colonies and inoculate them into the prepared wells.

**OPTIONAL** A1 wells of each plate can be left without any colony being inoculated as a negative control.

7. Secure the round-bottom plates in a plastic box affixed to a shaking platform inside an incubator. Remove the covers from the plates, seal the box with cling film, and incubate the bacteria overnight at 37 °C, 250 rpm.

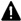
 **CRITICAL STEP** To prevent cross-contamination, keep the plates covered while securing them inside the box, and only remove the 96-well plate covers just before sealing the box with cling film.

8. The following day, prepare a new set of 96-well round-bottom plates with 200 μL of 2xTY-G. Transfer 5 μL from the overnight cultures into the new plates using a multichannel pipette.

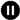
 **PAUSE STEP** The original 96-well plate with overnight-grown colonies is the ‘master plate.’ Remove 45 μL from the master plate to prevent expansion of the media during freezing, add 100 μL of 50% glycerol to achieve a 20% final glycerol concentration, and store at −80 °C for future use.

9. After 3 h of growth, add 50 μL of 2xTY-G containing 4 × 10^8^ cfu of helper phages to each well, gently mixing to ensure even distribution of the phages with the bacteria.
10. Incubate at 37 °C for 1 h without shaking to allow for helper phage infection. Then, centrifuge the plates at 4000× *g* for 10 min and discard the supernatant carefully to prevent cross-contamination between wells, by a single vigorous downward motion. Blot the plates on a paper towel and resuspend the bacterial pellets in 200 μL of 2xTY-I, ensuring thorough mixing.
11. Return the plates to the incubator and induce phage production overnight at 25 °C and 250 rpm.
12. Prior to leaving for the day, coat additional ELISA plates with control and target antigens using the previously optimized conditions. Prepare an equal number of plates for each set of monoclonal phages picked.

**OPTIONAL** To reduce the amount of target antigen used, half-volume microtiter plates can be utilized, which allows for the coating volume to be decreased from 100 μL to 50 μL.

13. The next day, wash the ELISA plates three times with PBS-T and block them with 200 μL of 5% milk in PBS-T with gentle shaking for 1 h.
14. During the blocking step, centrifuge the phage induction plates at 4000× *g* for 10 min. Transfer 60 μL of the supernatant to new round-bottom 96-well plates containing 180 μL of 5% milk in PBS-T.

**OPTIONAL** A larger volume (up to 80 μL) can be transferred to increase the volume in the next step, but care should be taken to avoid aspirating bacterial pellets. Also, consider the maximum volume capacity of the round-bottom plates.

15. After blocking, add 100 μL of the diluted phage solution to each corresponding well in the ELISA plates. Incubate for 1 h (or 2 h to increase the ELISA signal) at RT with gentle agitation.
16. Wash the plates three times with PBS-T, then add 100 μL of HRP-conjugated anti-M13 coat protein antibody, following the manufacturer’s dilution guidelines. Incubate for 1 h at RT with gentle shaking.
17. Wash the plate five times with PBS-T and add 100 μL of TMB substrate to develop the color. Stop the reaction with 50 μL of 1 M H_2_SO_4_ and quantify the signal of OD_450_ using a plate reader.
18. Normalize the ELISA results by subtracting the control antigen quantifications from those of the target antigen. Select colonies with normalized values exceeding a predetermined cutoff for further assays specific to the project.

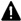
 **CRITICAL STEP** High ELISA signals, even after normalization, do not guarantee strong binding in other assays, such as FACS of Western blot using phages carrying scFv.  Therefore, it is recommended to use a low cutoff value, such as ‘top 33%’, for leniency in determining which colonies to further analyze.

19. Concurrently with hit selection, perform sequencing of the selected hits. This can be done by inoculating bacteria from the frozen master plate into 3 to 5 mL of LB media supplemented with ampicillin in a 14 mL tube for miniprep or colony PCR. Standard Sanger sequencing can then be carried out to identify the sequence of the hits, with primer details provided in Appendix A. Identify unique hits to proceed with further specific assays.

## 4. Expected Results

### 4.1. scFv Library Generation

**Figure 3:**
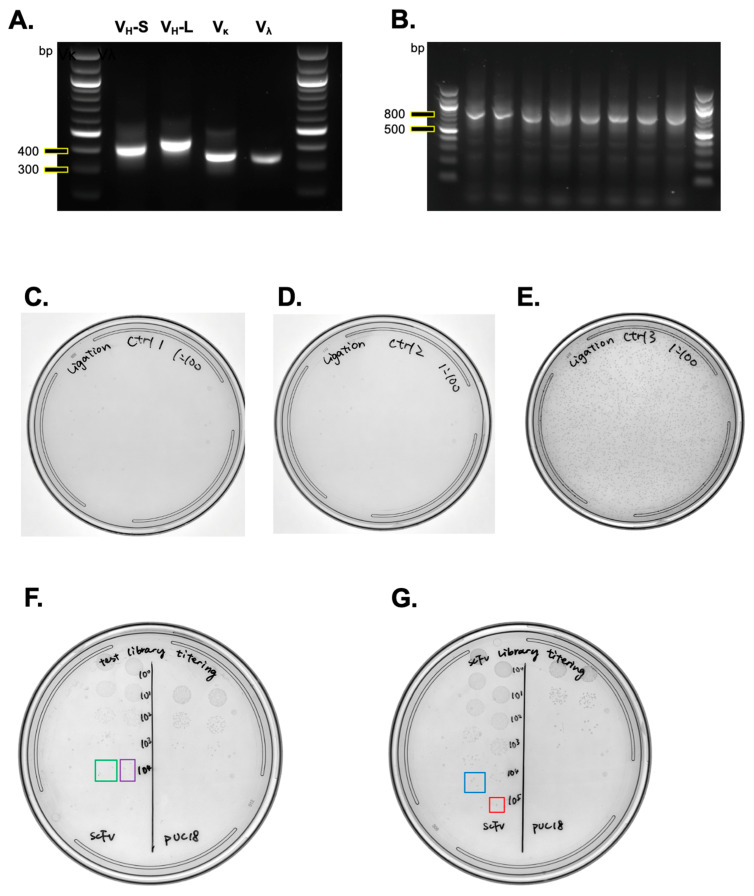
Expected results from scFv library generation. (**A**) Agarose gel electrophoresis of V_H_-S, V_H_-L, V_κ_, and V_λ_: This panel shows an example of V_H_-S, V_H_-L, V_κ_, and V_λ_ analyzed using 1.5% agarose gel electrophoresis. The expected sizes are approximately 400 bp for V_H_-S, 450 bp for V_H_-L, and 350 bp each for V_κ_ and V_λ._ (**B**) Random scFv fragments: Here, eight randomly selected scFv fragments are analyzed using 1.5% agarose gel electrophoresis. (**C**–**E**) Control ligation results: These panels depict control ligation results for ligation without ligase (**C**), without insert (**D**), and ligation with the vector and stuffer (**E**). (**F**) Test library generation: This panel demonstrates the result of the test library generation, highlighting two colonies in a green box and four colonies in a purple box. (**G**) Large-scale library generation: An example result of large-scale library generation, showing three colonies in a blue box and one colony in a red box.

Our example library was derived from the PBMCs of patients with an active viral infection. Total RNA was extracted and subsequently reverse-transcribed to generate cDNA. This cDNA served as the template for the amplification of the V regions. The amplified products of V_H_-S, V_H_-L, V_κ_, and V_λ_ were approximately 400 bp in size (Figure 3A), which were then precipitated and purified using gel extraction. The extracted V_H_ and V_L_ constructs were linked via secondary overlapping extension (SOE) PCR, resulting in major bands between 700 and 800 bp, varying based on the linker length (Figure 3B). The scFvs thus generated were electroporated into XL1-Blue competent cells. The test library displayed an estimate size of 1.8 × 10^7^ per ligation reaction, with minimal colony formation observed on control #1 (without ligase, Figure 3C) and control #2 plates (without insert, Figure 3D). In contrast, the control ligation involving a linearized vector and a stuffer insert resulted in numerous colonies, indicating successful ligation (Figure 3E). This suggests that the majority of the colonies were products of scFv ligated with the pComb3XSS vector.

The test library, when plated with a dilution series, yielded two and four colonies from duplicate inoculations. The size of the test library was calculated as follows:

$$\;Test\;library\;size\;from\;single\;ligation=\;\;\left(average\;count\;of\;colonies\right)\times dilution\;factor\times \frac{total\;volume\;of\;elctroporated\;competent\;cells}{inoculation\;volume}$$

Using our example (Figure 3F), the calculation was:

$$\frac{\left(2+4\right)}{2}\times 10^{4}\times \frac{3000\;\mu L}{5\;\mu L}=1.8\times 10^{7}$$

To ensure a library with higher diversity, we set a goal for the library size to exceed 1 × 10^8^ in the large-scale library generation. We conducted 20 ligation reactions and used a dilution series to determine the final library size (Figure 3G). The size of the final library was calculated as follows:

$$\frac{\left(3+1\right)}{2}\times 10^{5}\times \frac{20000\;\mu L}{5\;\mu L}=8\times 10^{8}$$

Upon confirming that the final library’s size exceeded our target of 1 × 10^8^, we proceeded to amplify the bacterial library on 245 mm plates. After amplification, the bacteria were scraped from the plates. We then prepared bacterial library stocks, aliquoting them into vials, each containing 5 × 10^9^ bacteria.

To assess the diversity of the generated library, we performed Sanger sequencing on randomly selected monoclonal bacterial colonies (as detailed in Table 5). This analysis revealed an even distribution among the V_H_-S-V_κ_, V_H_-L-V_κ_, V_H_-S-V_λ_, and V_H_-L-V_λ_ constructs. Such an even distribution is indicative of a well-representative library, encompassing a broad range of the targeted antibody repertoire.

### 4.2. Phage Display Affinity Selection and Antigen-Specific Antibody Development

**Figure 4:**
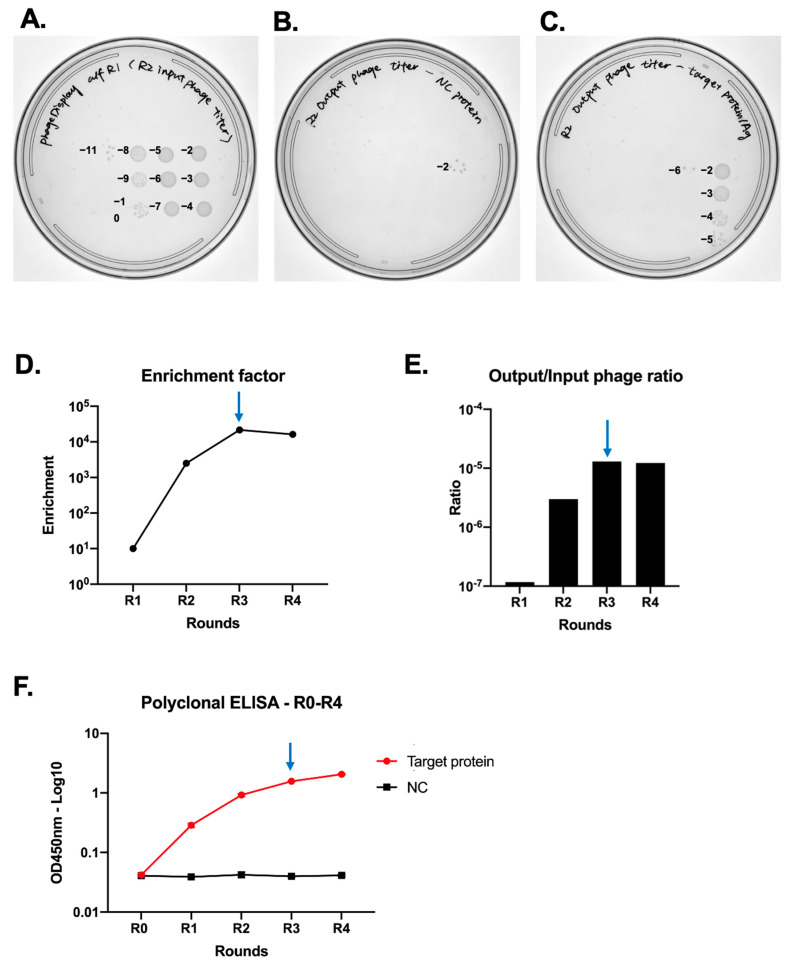
Expected results from phage display screening. (**A**) Input phage titration: This panel shows an example of titrating the input phages. (**B**) Control antigen output phage titration: This panel displays the results of titration for phages bound to the control antigen. (**C**) Target antigen output phage titration: This panel illustrates the results of titration for phages bound to the target antigen. (**D**–**F**) Enrichment analysis results: These panels present the outcomes of the enrichment analysis. Panel D shows the results of enrichment factor calculations; Panel E depicts the output/input phage titer calculations; and Panel F presents data from polyclonal ELISA, all as outlined in Section 3.7.3, “Enrichment analysis,” and Section 3.7.4, “Polyclonal ELISA.” Blue arrows in these panels indicate the recommended stopping point for affinity selection. R0, R1, R2, R3, and R4 represent phages rescued from the naïve scFv library and after rounds 1, 2, 3, and 4 of affinity selection, respectively.

Our phage display affinity selection process commenced with a bacterial library containing approximately 5 × 10^9^ bacteria. We initiated phage production by infecting the bacterial cells with 4 × 10^10^ cfu of helper phages. Once the phages were induced, they were precipitated using a PEG solution. Subsequently, the phages carrying scFv repertoires were serially diluted and applied to plates for titration prior to in vitro selection, as demonstrated in Figure 4A.

The quantification of phage titer was determined using the following formula:

$$\;Phage\;titer\;as\;cfu/\mathrm{mL}=\;\;count\;of\;colonies\times dillution\;factor\times \frac{total\;volume\;of\;infected\;bacteria}{volume\;of\;inoculated\;bacteria}$$

In our study (as illustrated in Figure 4A), the calculation was performed as follows:

$$6\times 10^{11}\times \frac{100\;\mu L\;}{5\;\mu L}=1.2\times 10^{13}\frac{cfu}{\mathrm{mL}}$$

Initially, 1 × 10^11^ cfu of induced phages displaying scFv were subjected to control antigen binding (input phage). The unbound phages were then transferred to wells coated with the target antigen. Bound phages for both control and target antigens were eluted separately (output phage, as shown in Figure 4B,C). We quantified the output phage titers from both the control antigen and target antigen across multiple rounds for enrichment analysis, as described in Section 3.7.3, “Enrichment analysis” (Figure 4D,E). Phages eluted from wells initially coated with target antigen were then amplified for subsequent rounds of affinity selection. Notably, both the enrichment factor and the output/input phage titer decreased (Figure 4D,E), while the polyclonal ELISA reached a plateau (Figure 4F). This indicated that maximum enrichment had been achieved, and further rounds of affinity selection were likely to result in the loss of target-specific phages. Therefore, we did not proceed with additional rounds of affinity selection.

**Figure 5:**
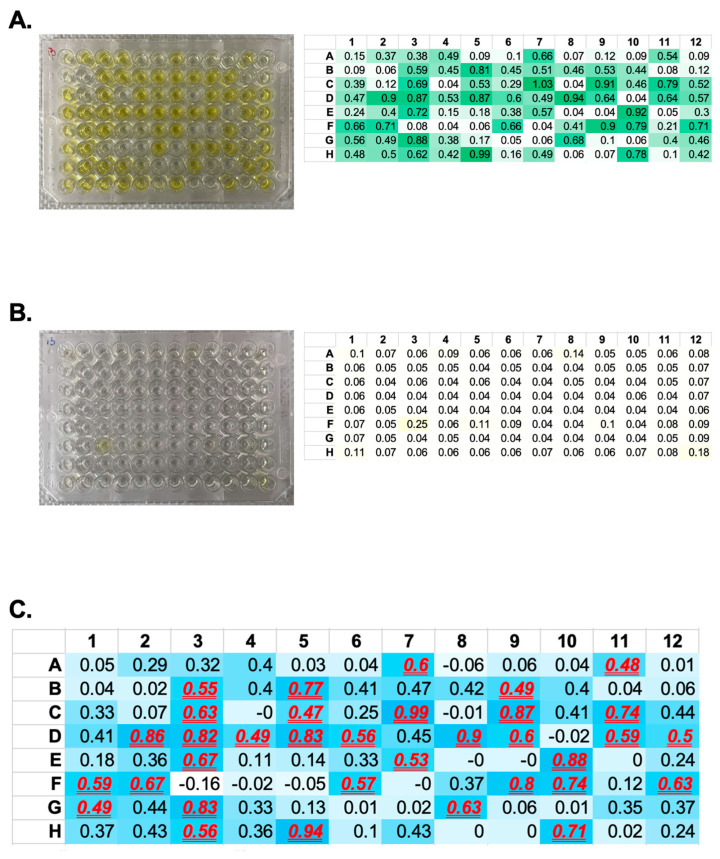
Expected results from monoclonal ELISA. (**A**,**B**) Monoclonal phage ELISA results: (**A**) represents the results using the target antigen as the coating antigen, while (**B**) shows the results with the control antigen used as the coating antigen. (**C**) Normalized monoclonal phage ELISA results: This panel illustrates the outcome of the normalized monoclonal phage ELISA. The normalization was performed by subtracting the OD_450_ values obtained from the control antigen ELISA (**B**) from those of the target antigen ELISA (**A**). The results that exceeded our predefined cutoff value (OD_450_ = 0.47) are highlighted in red, bold, and underlined font, indicating positive hits.

We performed serial dilutions of the bacterial stock from Round 3, as the product from the third round of affinity selection was expected to contain the most diverse anti-target antigen scFvs. Single bacterial colonies were then picked into 96-well plates. In our case, we selected a total of 14 plates (1330 single colonies, leaving the A1 well empty as a negative control). Following colony growth and phage induction in each well, the induced phages were used for binding to plates coated with both control antigen (Figure 5A) and target antigen (Figure 5B) in ELISA assays.

The normalized ELISA value was calculated as follows:

$$\;Normalized\;ELISA\;value\;\;={OD}_{450}\;from\;target\;protein\;ELISA-{OD}_{450}\;from\;control\;protein\;ELISA$$

We displayed the normalized ELISA values using a color gradient to visually highlight wells where the normalized ELISA exceeded an arbitrary cutoff value (Figure 5C).

So far, this protocol has been successfully employed to generate multiple pathogen-specific libraries and to screen for a variety of antigen-specific binders. Notably, the consistency of the data has been demonstrated across a diverse range of proteins. This includes applications in identifying viral surface glycoproteins for therapeutic antibody development, as well as transmembrane G-protein-coupled receptors (GPCRs) for structural analysis.

In summary, this paper presents a comprehensive and meticulous scFv library generation protocol, along with a detailed description of its subsequent phage display screening procedure. The methods outlined here have been shown to be effective and reproducible, offering significant potential for varied applications in the field of molecular biology and therapeutic development.

## 5. Reagents Setup

A 45% glucose solution: Slowly add glucose to 1 L of deionized water while stirring. Add only 50 g at a time until 450 g is added. Mildly heat the solution up to 50 °C or lower. Filter the solution through a 0.2 μm filter.

LB agar plate supplemented with 2% *w*/*v* glucose and 50 μg/mL ampicillin: Dissolve 20 g of LB broth powder in 900 mL of deionized water. After autoclaving and cooling the mixture to approximately 60 °C, add 44.4 mL of filtered 45% glucose solution and ampicillin to the final 50 μg/mL. Add autoclaved and deionized water to 1 L. For distribution, pour 20 mL into each 10 cm Petri dish and 150 mL into each 245 mm square plate.

H_2_SO_4_ ELISA stop solution: Carefully mix 10 mL of 100% H_2_SO_4_ into 177.7 mL of deionized water, ensuring to add the acid slowly and steadily into the water.

2xTY-Growth medium (2xTY with 2% *w*/*v* glucose and ampicillin, 2xTY-G): Dissolve 5 g NaCl, 10 g yeast extract, and 16 g bacto-tryptone in 900 mL of deionized water. After autoclaving, cool the solution to RT, then add 44.4 mL of filtered 45% glucose solution. Add autoclaved and deionized water to 1 L. Just before use, enrich the medium with ampicillin to a final concentration of 50 μg/mL.

2xTY-Induction (2xTY with ampicillin and kanamycin, 2xTY-I) medium: Dissolve 5 g NaCl, 10 g yeast extract, and 16 g bacto-tryptone in 1L of deionized water. After autoclaving, allow the medium to reach RT. Before use, fortify the medium with both ampicillin and kanamycin to reach a final concentration of 50 μg/mL each.

PEG solution (20% PEG-6000, 2.5 M NaCl): Dissolve 100 g of PEG-6000 and 73 g of NaCl in 500 mL of deionized water. Filter through a 0.2 μm filter for sterility.

PBS-T (0.05% Tween 20): Add 250 μL Tween 20 to 500 mL PBS.

AEBSF protease inhibitor: dissolve 4 mg of AEBSF in 1 mL of PBS and store aliquots at −20 °C for later use.

Trypsin stock solution (1 mg/mL): Dissolve 1 g of trypsin powder in 100 mL of PBS. Store this solution in aliquots at −20 °C. When needed, dilute the stock solution with PBS at a ratio of 1:4 to achieve a working trypsin solution of 0.25 mg/mL.

## Figures and Tables

**Table 1:** Composition of V, D, and J segments in the human genome [31].

	Heavy Chain (V_H_)	Light Chain (V_L_)	
		κ Light	λ Light
V segment	40	40	30
D segment	25	-	-
J segment	6	5	4

**Table 2:** Conditions for V gene amplification PCR.

Reaction Mixture		Touch Down PCR Condition	
Platinum Hot Start Taq 2× Master Mix	12.5 μL	94 °C	2 min
Autoclaved H_2_O	7 μL	94 °C	30 s ^β^
First-strand cDNA	0.5 μL	80 °C to 68 °C	30 s (−1 °C/cycle) ^β^
V region forward primer (10 μM) (V segment) ^α^	2.5 μL	72 °C	1.5 min ^β^
V region reverse primer (10 μM) (J segment) ^α^	2.5 μL	94 °C	30 s ^χ^
		50 °C	30 s ^χ^
		72 °C	1.5 min ^χ^
		72 °C	10 min
		105 °C	Lid temp

^α^ Total primer combinations: **V_H_**–**S**: 19 of V_H_ Forward (F′) primers × 6 of V_H_ Reverse (R′) primers carrying short linker = 114; **V_H_-L**: 19 of V_H_ F′ primers × 6 of V_H_ R′ primers carrying long linker = 114; **V_κ_** (light chain): 12 of V_K_ F′ primers × 5 of V_κ_ R′ primers with partial overlap with short linker = 60; **V_λ_** (light chain): 20 of V_λ_ F′ primers × 3 of V_λ_ R′ primers with partial overlap with long linker = 60. ^β^ Perform 12 cycles of PCR with conditions of 30 s at 94 °C, 1 °C reduction/cycle from 80 °C to 68 °C, followed by 1.5 min at 72 °C. ^χ^ Perform 22 cycles of PCR with conditions of 30 s at 94 °C, 30 s at 50 °C, 1.5 min at 72 °C.

**Table 3:** Conditions for SOE PCR.

Reaction Mixture		Touch Down PCR Condition	
Platinum Hot Start Taq 2× Master Mix	12.5 μL	94 °C	2 min
V_H_-short or V_H_-long	50 ng	94 °C	30 s ^β^
V_κ_ or V_λ_	50 ng	56 °C	30 s ^β^
SOE primer—F ^α^	2.5 μL	72 °C	30 s ^β^, **20 cycles**
		12 °C ^χ^	∞
SOE primer—R ^α^	2.5 μL	94 °C	30 s ^δ^
Autoclaved H_2_O	To 25 μL	68 °C	30 s ^δ^
		72 °C	1 min ^δ^
		72 °C	10 min
		105 °C	Lid temp

^α^ We recommend adding the primers after 20 PCR cycles to ensure adequate annealing of V_H_-V_L_ annealing. ^β^ Perform the initial 20 cycles at 94 °C for 30 s, and at 56 °C for 30 s to allow the overlapping sequences to anneal. ^χ^ At this step, remove the tubes from the thermocycler and add 2.5 μL of forward primer (F′) and 2.5 μL of reverse primer (R′). Subsequently, return the tubes to the thermocycler and proceed with the ‘next step.’ ^δ^ Perform an additional 15 cycles at 94 °C for 30 s, at 68 °C for 30 s, and at 72 °C for 1 min.

**Table 4:** Conditions for the T4 DNA ligase reaction.

Reaction Mixture	Control 1 (No Ligase)	Control 2 (No Insert)	Control 3 (with Stuffer Insert)	scFv	T4 DNA Ligase Reaction Condition	
T4 10× ligation buffer	2 μL	2 μL	2 μL	2 μL	16 °C	16 h
Digested scFv	-	-	-	70 ng	65 °C	10 min
Stuffer fragment	-	-	70 ng	-	4 °C	Hold
Digested pComb3XSS	140 ng	140 ng	140 ng	140 ng	105 °C	Lid temp
T4 ligase	-	1 μL (400 U)	1 μL (400 U)	1 μL (400 U)		
Autoclaved H_2_O	To 20 μL	To 20 μL	To 20 μL	To 20 μL		

**Table 5:** Library quality control (QC) sequence analysis. scFv constructs featuring V_H_-V_κ_ are denoted with an underlined font. V_H_-V_λ_ scFv constructs are indicated in bold font. scFvs incorporating long linkers are highlighted in grey. Those with short linkers are distinguished with a yellow highlight.

Sample ID	Heavy Chain V Segment Family	Heavy Chain J Segment Family	Light Chain V Segment Family	Light Chain J Segment Family	Sample ID	Heavy Chain V Segment Family	Heavy Chain J Segment Family	Light Chain V Segment Family	Light Chain J Segment Family	Sample ID	Heavy Chain V Segment Family	Heavy Chain J Segment Family	Light Chain V Segment Family	Light Chain J Segment Family
** QC1 **	HUVH3E	Hujh3-S	HUVK6B	hujk1	** QC11 **	HUVH6	Hujh6b-L	HUVK1C	hujk2	**QC21**	HUVH3C	hujh45	HUVL2C	hujl23
** QC2 **	HUVH3B	Hujh45-S	HUVK3B	hujk2	**QC12**	HUVH1A	Hujh2-L	HUVL3C	hujl23	**QC22**	HUVH1A	hujh6b	HUVL2B	hujl23
**QC3**	HUVH1A	Hujh2-S	HUVL2B	hujl1	** QC13 **	HUVH1C	Hujh6b-L	HUVK3C	hukj2	** QC23 **	HUVH4C	hujh45	HUVK1C	hujk2
**QC4**	HUVH3C	Hujh1-S	HUVL6	hujl1	** QC14 **	HUVH4A	Hujh3-S	HUVK3A	hujk4	** QC24 **	HUVH1A	hujh45	HUVK1B	hujk3
** QC5 **	HUVH1C	Hujh2-L	HUVK1B	hujk1	** QC15 **	HUVH1C	Hujh1-L	HUVK3A	hujk2	**QC25**	HUVH1A	Hujh1	HUVL2C	hujl1
**QC6**	HUVH1A	Hujh3-L	HUVL5A9	hujl1	** QC16 **	HUVH4C	Hujh2-L	HUVK1A	hujk4	**QC26**	HUVH3E	Hujh3	HUVL8	hujl23
** QC7 **	HUVH1C	Hujh3-S	HUVK2A	hujk1	**QC17**	HUVH4D	Hujh2-S	HUVL1A	hujl1	**QC27**	HUVH1C	Hujh3	HUVL6	hujl1
** QC8 **	HUVH1C	Hujh45-L	HUVK3B	hujk3	** QC18 **	HUVH4A	Hujh6a-S	HUVK2B	hujk1	**QC28**	HUVH4B	hujh6b	HUVL1B	hujl23
**QC9**	HUVH4D	Hujh45-L	HUVL10	hujl1	**QC19**	HUVH4B	Hujh6a-S	HUVL2A	hujl7	** QC29 **	HUVH2A	Hujh1	HUVK1A	hujk3
**QC10**	HUVH1B	Hujh45-S	HUVL10	hujl1	** QC20 **	HUVH1A	hujh2	HUVK6A	hujk4					

## Data Availability

No additional data were created or analyzed in this study. Data sharing is not applicable to this article. The authors can be contacted for any further information regarding the data within the article.

## Appendix A. Primers for scFv Library Generation

**V_H_ F′ primers (nineteen in total)**

V_H_ F1:

5′-GGTTTCGCTACCGTGGCCCAGGCGGCCATGGCCCAGGTGCAGCTGGTGCAGTCTGG-3′

VH F2:

5′-GGTTTCGCTACCGTGGCCCAGGCGGCCATGGCCCAGGTYCAGCTKGTGCAGTCTGG-3′

VH F3:

5′-GGTTTCGCTACCGTGGCCCAGGCGGCCATGGCCCAGGTCCAGCTGGTACAGTCTGG-3′

VH F4:

5′-GGTTTCGCTACCGTGGCCCAGGCGGCCATGGCCCARATGCAGCTGGTGCAGTCTGG-3′

VH F5:

5′-GGTTTCGCTACCGTGGCCCAGGCGGCCATGGCCCAGGTSCAGCTGGTGCARTCTGG-3′

VH F6:

5′-GGTTTCGCTACCGTGGCCCAGGCGGCCATGGCCCAGRTCACCTTGAAGGAGTCTGG-3′

VH F7:

5′-GGTTTCGCTACCGTGGCCCAGGCGGCCATGGCCCAGGTCACCTTGAGGGAGTCTGG-3′

VH F8:

5′-GGTTTCGCTACCGTGGCCCAGGCGGCCATGGCCSAGGTGCAGCTGGTGGAGTCTGG-3′

VH F9:

5′-GGTTTCGCTACCGTGGCCCAGGCGGCCATGGCCGAGGTGCAGCTGTTGGAGTCTGG-3′

VH F10:

5′-GGTTTCGCTACCGTGGCCCAGGCGGCCATGGCCGAGGTGCAGCTGGTGGAGWCYGG-3′

VH F11:

5′-GGTTTCGCTACCGTGGCCCAGGCGGCCATGGCCGAAGTGCAGCTGGTGGAGTCTGG-3′

VH F12:

5′-GGTTTCGCTACCGTGGCCCAGGCGGCCATGGCCCAGGTACAGCTGGTGGAGTCTGG-3′

VH F13:

5′-GGTTTCGCTACCGTGGCCCAGGCGGCCATGGCCCAGSTGCAGCTGCAGGAGTCGGG-3′

VH F14:

5′-GGTTTCGCTACCGTGGCCCAGGCGGCCATGGCCCAGGTGCAGCTACAGCAGTGGGG-3′

VH F15:

5′-GGTTTCGCTACCGTGGCCCAGGCGGCCATGGCCCAGCTGCAGCTGCAGGAGTCCGG-3′

VH F16:

5′-GGTTTCGCTACCGTGGCCCAGGCGGCCATGGCCCAGGTGCAGCTACAACAGTGGGG-3′

VH F17:

5′-GGTTTCGCTACCGTGGCCCAGGCGGCCATGGCCGARGTGCAGCTGGTGCAGTCTGG-3′

VH F18:

5′-GGTTTCGCTACCGTGGCCCAGGCGGCCATGGCCCAGGTACAGCTGCAGCAGTCAGG-3′

VH F19:

5′-GGTTTCGCTACCGTGGCCCAGGCGGCCATGGCCCAGGTGCAGCTGGTGCAATCTGG-3′

**VH R′ short linker primers (VH-S R′, six in total)**

VH-S R1:

5′-GGAAGATCTAGAGGAACCACC TGAGGAGACGGTGACCAGGGTGCCCTG-3′

VH-S R2:

5′-GGAAGATCTAGAGGAACCACC TGAGGAGACAGTGACCAGGGTGCCACG-3′

VH-S R3:

5′-GGAAGATCTAGAGGAACCACC TGAAGAGACGGTGACCATTGTCCCTTG-3′

VH-S R4:

5′-GGAAGATCTAGAGGAACCACC TGAGGAGACGGTGACCAGGGTYCCYTG-3′

VH-S R5:

5′-GGAAGATCTAGAGGAACCACC TGAGGAGACGGTGACCGTGGTCCCTTG-3′

VH-S R6:

5′-GGAAGATCTAGAGGAACCACC TGAGGAGACGGTGACCGTGGTCCCTTT-3′

**VH R′ long linker (VH-L R′, six in total)**

VH-L R′1:

5′-GGAAGATCTAGAGGAACCACCCCCACCACCGCCCGAGCCACCGCCACCAGAGGATGAGGAGACGGTGACCAGGGTGCCCTG-3′

VH-L R′2:

5′-GGAAGATCTAGAGGAACCACCCCCACCACCGCCCGAGCCACCGCCACCAGAGGA TGAGGAGACAGTGACCAGGGTGCCACG-3′

VH-L R′3:

5′-GGAAGATCTAGAGGAACCACCCCCACCACCGCCCGAGCCACCGCCACCAGAGGA TGAAGAGACGGTGACCATTGTCCCTTG-3′

VH-L R′4:

5′-GGAAGATCTAGAGGAACCACCCCCACCACCGCCCGAGCCACCGCCACCAGAGGA TGAGGAGACGGTGACCAGGGTYCCYTG-3′

VH-L R′5:

5′-GGAAGATCTAGAGGAACCACCCCCACCACCGCCCGAGCCACCGCCACCAGAGGA TGAGGAGACGGTGACCGTGGTCCCTTG-3′

VH-L R′6:

5′-GGAAGATCTAGAGGAACCACCCCCACCACCGCCCGAGCCACCGCCACCAGAGGA TGAGGAGACGGTGACCGTGGTCCCTTT-3′

**Vκ (kappa light chain) F′ (twelve in total)**

Vκ F′1:

5′-GGTGGTTCCTCTAGATCTTCC GACATCCAGWTGACCCAGTCTCC-3′

Vκ F′2:

5′-GGTGGTTCCTCTAGATCTTCC GCCATCCRGWTGACCCAGTCTCC-3′

Vκ F′3:

5′-GGTGGTTCCTCTAGATCTTCC GTCATCTGGATGACCCAGTCTCC-3′

Vκ F′4:

5′-GGTGGTTCCTCTAGATCTTCC GATATTGTGATGACCCAGACTCC-3′

Vκ F′5:

5′-GGTGGTTCCTCTAGATCTTCC GATRTTGTGATGACTCAGTCTCC-3′

Vκ F′6:

5′-GGTGGTTCCTCTAGATCTTCC GAAATTGTGTTGACRCAGTCTCC-3′

Vκ F′7:

5′-GGTGGTTCCTCTAGATCTTCC GAAATAGTGATGAYGCAGTCTCC-3′

Vκ F′8:

5′-GGTGGTTCCTCTAGATCTTCC GAAATTGTAATGACACAGTCTCC-3′

Vκ F′9:

5′-GGTGGTTCCTCTAGATCTTCC GACATCGTGATGACCCAGTCTCC-3′

Vκ F′10:

5′-GGTGGTTCCTCTAGATCTTCC GAAACGACACTCACGCAGTCTCC-3′

Vκ F′11:

5′-GGTGGTTCCTCTAGATCTTCC GAAATTGTGCTGACTCAGTCTCC-3′

Vκ F′12:

5′-GGTGGTTCCTCTAGATCTTCC GATGTTGTGATGACACAGTCTCC-3′

**Vκ (kappa light chain) R′ (five in total)**

Vκ R′1:

5′-ATGGTGATGGTGATGGTGCTGGCCGGCCTGGCCACGTTTGATTTCCACCTTGGTCCC-3′

Vκ R′2:

5′-ATGGTGATGGTGATGGTGCTGGCCGGCCTGGCCACGTTTGATCTCCAGCTTGGTCCC-3′

Vκ R′3:

5′-ATGGTGATGGTGATGGTGCTGGCCGGCCTGGCCACGTTTGATATCCACTTTGGTCCC-3′

Vκ R′4:

5′-ATGGTGATGGTGATGGTGCTGGCCGGCCTGGCCACGTTTGATCTCCACCTTGGTCCC-3′

Vκ R′5:

5′-ATGGTGATGGTGATGGTGCTGGCCGGCCTGGCCACGTTTAATCTCCAGTCGTGTCCC-3′

**V_λ_ (lambda light chain) F′ (twenty in total)**

Vλ F′1:

5′-GGTGGTTCCTCTAGATCTTCC CAGTCTGTGCTGACTCAGCCACC-3′

Vλ F′2:

5′-GGTGGTTCCTCTAGATCTTCC CAGTCTGTSSTGACGCAGCCGCC-3′

Vλ F′3:

5′-GGTGGTTCCTCTAGATCTTCC CAGTCTGTGTTGACGCAGCCGCC-3′

Vλ F′4:

5′-GGTGGTTCCTCTAGATCTTCC CAGTCTGCCCTGACTCAGCCTCC-3′

Vλ F′5:

5′-GGTGGTTCCTCTAGATCTTCC CAGTCTGCCCTGACTCAGCCTCG-3′

Vλ F′6:

5′-GGTGGTTCCTCTAGATCTTCC CAGTCTGCCCTGACTCAGCCTGC-3′

Vλ F′7:

5′-GGTGGTTCCTCTAGATCTTCC TCCTATGWGCTGACTCAGCCACC-3′

Vλ F′8:

5′-GGTGGTTCCTCTAGATCTTCC TCCTATGAGCTGACTCAGCCACT-3′

Vλ F′9:

5′-GGTGGTTCCTCTAGATCTTCC TCTTCTGAGCTGACTCAGGACCC-3′

Vλ F′10:

5′-GGTGGTTCCTCTAGATCTTCC TCCTATGAGCTGACACAGCYAYC-3′

Vλ F′11:

5′-GGTGGTTCCTCTAGATCTTCC TCCTATGAGCTGATGCAGCCAC-3′

Vλ F′12:

5′-GGTGGTTCCTCTAGATCTTCC CTGCCTGTGCTGACTCAGCCCCCGT-3′

Vλ F′13:

5′-GGTGGTTCCTCTAGATCTTCC CAGCCTGTGCTGACTCAATCATC-3′

Vλ F′14:

5′-GGTGGTTCCTCTAGATCTTCC CAGCTTGTGCTGACTCAATCGCC-3′

Vλ F′15:

5′-GGTGGTTCCTCTAGATCTTCC CAGCCTGTGCTGACTCAGCCAYC-3′

Vλ F′16:

5′-GGTGGTTCCTCTAGATCTTCC CAGGCTGTGCTGACTCAGCCGKC-3′

Vλ F′17:

5′-GGTGGTTCCTCTAGATCTTCC AATTTTATGCTGACTCAGCCCCA-3′

Vλ F′18:

5′-GGTGGTTCCTCTAGATCTTCC CAGRCTGTGGTGACTCAGGAGCC-3′

Vλ F′19:

5′-GGTGGTTCCTCTAGATCTTCC CAGACTGTGGTGACCCAGGAGCC-3′

Vλ F′20:

5′-GGTGGTTCCTCTAGATCTTCC CAGGCAGGGCTGACTCAGCCACC-3′

**V_λ_ (lambda light chain) R′ (three in total)**

Vλ R′1:

5′-ATGGTGATGGTGCTGGCCGGCCTGGCC ACCTAGGACGGTGACCTTGGTCCCAG-3′

Vλ R′2:

5′-ATGGTGATGGTGCTGGCCGGCCTGGCC ACCTAGGACGGTCAGCTTGGTCCCTC-3′

Vλ R′3:

5′-ATGGTGATGGTGCTGGCCGGCCTGGCC ACCGAGGACGGTCAGCTGGGTGCCTC-3′

**SOE—Secondary Overlapping Extension (two):**

SOE F′:

5′-GAGGAGGAGGAGGAGGGTTTCGCTACCGTGGCCCAGGCGGCC-3′

SOE R′:

5′-GAGGAGGAGGAGGAGATGGTGATGGTGCTGGCCGGCCTGGCC-3′

**Recommended sequencing primer (for pComb3XSS phagemid):**

F′:

5′-ATTTAAAATGAAAAAGACAGCTATCGCG-3′

R’:

5′-GCTTAAATTAATTAGCTAGCTTAAGACTCC-3′
